# The Cognitive Underpinnings of Multiply-Constrained Problem Solving

**DOI:** 10.3390/jintelligence9010007

**Published:** 2021-02-01

**Authors:** Derek M. Ellis, Matthew K. Robison, Gene A. Brewer

**Affiliations:** 1 Department of Psychology, Arizona State University, Tempe, AZ 85281, USA; dmellis2@asu.edu; 2 Department of Psychology, University of Texas Arlington, Arlington, TX 76019, USA; matthew.robison@uta.edu

**Keywords:** multiply-constrained problem solving, individual differences, insight

## Abstract

Individuals encounter problems daily wherein varying numbers of constraints require delimitation of memory to target goal-satisfying information. Multiply-constrained problems, such as the compound remote associates, are commonly used to study this type of problem solving. Since their development, multiply-constrained problems have been theoretically and empirically related to creative thinking, analytical problem solving, insight problem solving, and a multitude of other cognitive abilities. In the present study, we empirically evaluated the range of cognitive abilities previously associated with multiply-constrained problem solving to assess common versus unique predictive variance (i.e., working memory, attention control, episodic and semantic memory, and fluid and crystallized intelligence). Additionally, we sought to determine whether problem-solving ability and self-reported strategy adoption (analytical or insightful) were task specific or task general through the use of novel multiply-constrained problem-solving tasks (TriBond and Location Bond). Performance across these tasks was shown to be domain general, solutions derived through insightful strategies were more often correct than those derived through analytical strategies, and crystallized intelligence was the sole cognitive ability that provided unique predictive value after accounting for all other abilities.

## 1. The Cognitive Underpinnings of Multiply-Constrained Problem Solving

Humans possess an incredible ability to target remote information stored in semantic memory even when provided with only minimal cues to guide their search. For example, consider participating on the game show *Jeopardy!* where contestants are provided with an answer and their goal is to find the specific question that generated that answer. To the naive viewer, this may seem like a nearly impossible problem to solve. However, contestants can use certain cues to delimit their search of memory. Specifically, the answers all come from a common category which narrows the search to a specific domain. Additionally, contestants’ responses are almost exclusively limited to “Who is/are” or “What is/are,” which means that dates are more often clues than answers. Lastly, the answer itself provides the final narrowing. *Jeopardy!* questions, such as the one above, can alternatively be classified as a multiply-constrained problem. More importantly, individuals engage in multiply-constrained problem solving every day, not just when an answer to a trivia questions needs to be retrieved. Specifically, when a doctor is treating a patient with an unknown ailment, the doctor will identify different symptoms, such as cough, runny nose, and elevated temperature, to eliminate unlikely or incorrect diagnoses and to eventually arrive at a correct diagnosis (i.e., common cold). Similarly, choosing a restaurant with a group of friends can be a multiply-constrained problem. The constraints in this situation are dietary limitations, location, and budget.

The *Jeopardy!* example highlights a type of convergent or multiply-constrained problem. While *Jeopardy!* questions have certainly been used in the classroom (Rotter 2004), in the laboratory a more commonly used set of multiply-constrained problems are the Compound Remote Associates Test. The Remote Associates Test, originally developed by Mednick (1962), requires an individual to search through memory for a target word (“ice”) that is semantically related to three cues (“cream, skate, water”). These problems were later adapted such that the target is paired with each cue to form a compound word or phrase (Bowden and Jung-Beeman 2003). Furthermore, the *Jeopardy!* example highlights possible underlying cognitive processes that lead to successful problem solving, as well as possible sources of interference in problem solving ability. Specifically, an individual’s ability to maintain control of attention in the face of irrelevant distractors and having been exposed to the correct information and actually having that information stored in memory are all possible sources of variability in multiply-constrained problem solving. Therefore, the purpose of this experiment was to determine how individual differences in working memory capacity, attention control, long-term memory, and fluid and crystallized intelligence predict performance in multiply-constrained problem solving.

### 1.1. Multiply-Constrained Problem Solving & Strategies

When an individual attempts to solve a multiply-constrained problem they may employ a strategy, and the two most commonly reported strategies are analytical (sometimes referred to simply as strategy; Zedelius and Schooler 2015) and insight. The analytical approach is defined as a stepwise approach like one would employ while solving a math problem. For a compound remote associates (CRA) problem the analytical approach would involve systematically generating and testing possible solutions against each cue word. Conversely, the insight strategy is exemplified by the “A-ha” moment where the solution appears to arise spontaneously (see (Weisberg 2015) for a review). We use the term strategy in order to be consistent with published research on this topic. However, our usage of the term strategy in this paper simply denotes the participants’ assessment of their subjective experience of discovering the solution to each problem they attempt to solve and not necessarily their approach to solving the problem. Although some have found that accuracy for analytical responses is better than insight (Chuderski and Jastrzebski 2018), the more consistent finding is that solutions reported to occur via insight are more often correct than solutions reported to occur via analytical strategy (Chein and Weisberg 2014; Salvi et al. 2016; Zedelius and Schooler 2015).

Given the way that the two strategies (analytical & insight) are conceptualized it is theoretically possible that different cognitive abilities will better support different strategies. Specifically, given the need to maintain the cues and retrieved responses during problem solving, working memory and attention control may better account for performance related to analytically retrieved responses. Specific to working memory there are inconsistent findings with regards to its role in problem solving. Some have found working memory to be correlated with problem solving (Chein and Weisberg 2014; Ellis and Brewer 2018), but others have found it to interfere (Ricks et al. 2007). Conversely, given the spontaneous retrieval of responses characterized by insight responses cognitive abilities related to memory retrieval and fluid intelligence may account for more variance (see (Wiley and Jarosz 2012a) for a review). Lastly, crystallized intelligence may predict both analytical and insight responses as the probability of retrieving a correct response from memory if it is not already in memory is low to nonexistent. As will be reviewed, prior studies have investigated individual differences in problem solving and relations with various cognitive abilities. However, much less research has examined individual differences in the strategies applied to solving multiply-constrained problems. 

### 1.2. Working Memory Capacity, Attention Control, & Multiply-Constrained Problem Solving

Attentional focus has been theorized as one key source of variation in problem solving performance (Wiley and Jarosz 2012a). Initial work by Lavric et al. (2000) identified that individual differences in WMC were predictive of both creative (which the CRA are thought to measure) and analytical problem solving. More recently, Chein and Weisberg (2014) provided further evidence that WMC correlates with multiply-constrained problem-solving ability, as measured by the CRA (see also Ellis and Brewer 2018; Lee and Therriault 2013; Ricks et al. 2007). Individual differences in working memory capacity are thought to arise due to differences in attention control, primary (or short-term) memory capacity, and cue-dependent retrieval from secondary (or long-term) memory (Unsworth 2016). Specifically, the focusing of attention allows an individual to actively search memory for possible solutions, resist distracting information, and let incorrect solutions decay (i.e., to reduce interference from previously generated but incorrect targets; Moss et al. 2011). However, there is some evidence that distractibility (Kim et al. 2007) or intoxication (Benedek et al. 2017; Jarosz et al. 2012) can aid performance by augmenting attention control functioning.

Given the relation between WMC and attention control (Engle 2002), it is possible that individual differences in WMC and attention control will account for a portion of the variance in multiply-constrained problem solving. For this experiment, we have chosen tasks that evaluate the different subcomponents of an individual’s attentional abilities using specifically the Stroop, Antisaccade, and Psychomotor Vigilance (PVT) tasks. Performance on the Stroop is related to goal maintenance (Kane and Engle 2003). Antisaccade performance is related to the ability to resist attention-capturing stimuli (Kane et al. 2001; Unsworth et al. 2004). Specifically, the Stroop and Antisaccade are both measures of attentional restraint, while the PVT captures an individual’s ability to sustain attention for periods of time and limit the number of executive control failures (Dinges and Powell 1985; Unsworth et al. 2010). Thus, we selected a set of attention tasks that can measure a broad goal-maintenance ability. All these tasks require the consistent maintenance and execution of a task goal in the face of potent internal and external distractors.

### 1.3. Memory and Multiply-Constrained Problem Solving

Recent work by Smith et al. (2013) and Davelaar (2015) highlights the role of semantic memory search operations during CRA problem solving (see also Smith and Huber 2015). These researchers examined whether CRA search behavior is similar to other semantic search tasks, such as category fluency. In a category fluency task, participants are asked to retrieve as many exemplars as possible given a specific category (e.g., animals). Both CRA and fluency tasks require retrieval of exemplars from memory. However, in the CRA, there is only one correct target, whereas in a fluency task there are many correct targets. When an individual completes a fluency task, they often cluster groups of responses together (Buschke 1977). For example, when given the category of animals, an individual will often choose a subcategory, such as aquatic animals, and provide several exemplars in rapid succession (Troyer et al. 1997), before switching to a different subcategory.

In many CRA experiments, the participant is only allowed to enter a single response for each problem, but others have allowed for multiple responses. For example, Davelaar (2015) used an externalized response procedure. In this procedure, the participant is asked to enter any potential answers that they generate during the problem-solving period for each problem and then ultimately say which answer that they believe is the correct answer. This externalized procedure allows the researcher to examine the participant’s semantic search path and the related associative distances between subsequent generated responses. Davelaar’s examination of responses during CRA problem solving found a clustering of responses similar to what is often found in a fluency task. However, recent findings call to question whether the results Smith et al. (2013) and Davelaar (2015) were an artifact caused by the externalized response procedure, leading problem solvers to utilize a serial search process rather than parallel (Howard et al. 2020). Additional individual differences work has identified that performance on fluency tasks is positively related with CRA performance (Lee and Therriault 2013). Given the relation between semantic fluency and CRA performance, along with the theoretical role that semantic search plays in solving CRA problems, an individual’s ability to effectively search semantic memory should be related to their problem-solving ability. While semantic retrieval abilities are related to both WMC and multiply-constrained problem solving, it must be noted that fluency tasks represent only a single type of memory retrieval. Other commonly used tasks such as Cued Recall, source monitoring, and Delayed Free Recall, which involve episodic retrieval mechanisms, also correlate with WMC (Unsworth and Engle 2007; Unsworth and Spillers 2010; Unsworth et al. 2010), attention control (Hutchinson and Turk-Brown 2012), semantic memory (Graham et al. 2000), and intelligence (Unsworth et al. 2009). Thus, the ability to retrieve answers from memory is necessary to solve multiply-constrained problems and may account for some of the shared variance among WMC, attention control, and multiply-constrained problem solving. Memory retrieval could provide unique predictive value above and beyond other cognitive abilities. More specifically, memory retrieval may account for differences in the rate at which correct solutions are retrieved, as our measures of memory retrieval may provide better estimates of accessibility rather than availability.

### 1.4. Intelligence and Multiply-Constrained Problem Solving

Lee and Therriault (2013) found that general knowledge predicted problem-solving ability. Similarly, reasoning ability, as measured by tasks such as Raven’s Progressive Matrices and Weschler Abbreviated Scale of Intelligence, correlates with problem-solving ability (Chuderski and Jastrzebski 2018; Kane et al. 2004). Importantly, it is fairly well established that problem solving correlates more strongly with measures of general knowledge than reasoning (Harris 2003; Lee and Therriault 2013). For a given problem, there is some fundamental knowledge one must have in order to solve it. For example, if given the CRA cues “cream, skate, water”, in order to be able to solve the problem, you would have to know at least these two things, (1) that “ice” is a word, (2) that “ice” forms a compound word or phrase with at least one of the cues. Therefore, knowledge of the target must serve as a limiting factor in problem solving. For the commonly used CRA problems, knowledge of cues and targets is often assumed to be evenly distributed as the words are commonly used, however distant the associations between cues and targets may be. In contrast to our long-term memory measures, the tasks used to measure crystallized intelligence provide better estimates of availability over accessibility of knowledge stored in memory.

### 1.5. The Current Study

Recently, there have been calls for more research focused on fundamental processes and abilities related to creativity and multiply-constrained problem solving (Benedek et al. 2012; Benedek and Fink 2019; Cortes et al. 2019; Dietrich 2019). Currently, the predominant theory is that working memory and associated attention and inhibitory processes are the most likely predictors of problem-solving ability (see Wiley and Jarosz 2012a). Attention control is needed in problem solving to generate possible solutions, ignore distraction, and inhibit previously retrieved solutions. However, other possible predictors are semantic memory and episodic memory. Work by Smith et al. (2013) and Davelaar (2015) highlight different search procedures and put forth their arguments for the most effective strategies. However, memory retrieval processes may not be a unique predictor as Unsworth et al. (2013) have shown that WMC is related to number of retrieved items and the use of effective retrieval strategies. Being able to generate more possible solutions should improve the odds of finding the target of multiply-constrained problems, such as the CRA, given that targets tend to be weakly related to the cues. Therefore, for the current experiment, we seek to use individual differences as a crucible to examine how working memory, attention control, memory retrieval, knowledge, and fluid reasoning are predictive of multiply-constrained problem-solving ability (Underwood 1975). While others have examined multiply-constrained problem-solving ability and possible predictors of performance, none have evaluated the range of cognitive abilities present in this experiment.

Participants completed multiple measures of multiply-constrained problem solving, WMC, attention control, long-term memory (episodic and semantic), and intelligence (crystallized and fluid). To better understand the role of these cognitive abilities in multiply-constrained problem solving, we adopted two additional remote associate tasks, TriBond and Location Bond (LocBond). The addition of these two tasks will allow us to better understand whether multiply-constrained problem solving is a general cognitive ability and whether the process by which individuals arrive at solutions to these problems is task specific or domain general. With these data, we sought to answer four key research questions, (1) do answers derived from analytical processes differ from those found through insight processes at the within-subject level? (2) Do different multiply-constrained problem-solving abilities represent individual task performance or a domain-general ability? (3) Are analytical and insight processes in multiply-constrained problem solving domain general or task specific? (4) Which, if any, of the cognitive abilities uniquely predict multiply-constrained problem-solving ability? Given the importance of accessibility and availability of information in memory, we analyzed conditionalized (i.e., analytical and insight) accuracy. Moreover, models were generated using response time for correct responses not conditionalized by strategy reported.

## 2. Method

### Participants and Design

Previous individual difference research on multiply-constrained problem solving had a sample size of 413 (Lee and Therriault 2013) participants. Therefore, a target sample size of at least 400 participants was established. Four hundred and ninety-one participants were recruited from the Arizona State University participant pool and received course credit for their participation. Prior to all statistical tests and modeling, the data were screened for outliers. First, individuals who failed to complete both days or who noted speaking English as a second language were removed from the data set as to not influence outlier detection. Second, all dependent measures were plotted and participants whose data were marked as repeatedly having outlying performance were removed from future analyses. Eight participants were removed for English not being their primary language, six were removed for participating in a manner nonconductive to accurate data collection (e.g., button mashing), two were removed for speed and accuracy errors during working memory tasks, three were removed due to being statistical outliers on working memory tasks (i.e., mean accuracy ± 3 SD of mean), four were removed for being statistical outliers on attention-based measures (i.e., average response times ± 3 SD of mean), and ten were removed for performing the Stroop task incorrectly (i.e., never identifying an incongruent trial). Therefore, the final data set includes 459 participants. Participants completed all experimental tasks across two separate group laboratory sessions lasting approximately two hours per day. Participants completed four working memory tasks, three attention control tasks, three long-term memory tasks, three semantic fluency tasks, three general knowledge tasks, three fluid intelligence tasks, and three multiply-constrained problem-solving tasks^1^.

## 3. Materials

**Demographics.** Participants were asked to provide general demographic information, such as age, whether they were a native English speaker or not, and if they are not a native English speaker, at what age they learned English. The Demographics also included the 10-item personality inventory which measured an individual’s openness, conscientiousness, emotional stability, agreeableness, and extraversion. Additionally, we evaluated an individual’s self-discipline, internal motivation, and external motivation.

**Reading Span.** Participants were required to read sentences while trying to remember a set of unrelated letters (F, H, J, K, L, N, P, Q, R, S, T, and Y). For this task, participants read a sentence and determined whether the sentence made sense or not (e.g., “The prosecutor’s dish was lost because it was not based on fact?”). Half of the sentences made sense while the other half did not. Nonsense sentences were made by simply changing one word (e.g., “dish” from “case”) from an otherwise normal sentence. Participants were required to read the sentence and to indicate whether it made sense or not. After participants gave their response, they were presented with a letter for 1 s. At recall, letters from the current set were recalled in the correct order by clicking on the appropriate letters. There were three trials of each list length with list length ranging from 3–7. The dependent measure was the number of correct items in the correct position.

**Operation Span.** Participants were required to solve a series of math operations while trying to remember the same set of unrelated letters as in the Reading Span. Participants were required to solve a math operation, and after solving the operation they were presented with a letter for 1 s. Immediately after the letter was presented the next operation was presented. Three trials of each list length (3–7) were presented, with the order of list length varying randomly. At recall, letters from the current set were recalled in the correct order by clicking on the appropriate letters (see Unsworth et al. 2005 for more details). Participants received three sets (of list length two) of practice. For all of the span measures, items were scored if the item was correct and in the correct position. The same scoring procedure as in the Reading Span was used.

**Symmetry Span.** In this task, participants were required to recall sequences of red squares within a matrix while performing a symmetry-judgment task. In the symmetry-judgment task, participants were shown an 8 × 8 matrix with some squares filled in black. Participants decided whether the design was symmetrical about its vertical axis. The pattern was symmetrical half of the time. Immediately after determining whether the pattern was symmetrical, participants were presented with a 4 × 4 matrix with one of the cells filled in red for 650 ms. At recall, participants recalled the sequence of red-square locations in the preceding displays, in the order they appeared, by clicking on the cells of an empty matrix. There were three trials of each list length, with list length ranging from 2–5. The same scoring procedure as in the Reading Span was used.

**Rotation Span.** The automated Rotation Span (Harrison et al. 2013) consists of to-be-remembered items that are a sequence of long and short arrows, radiating from a central point.

The processing task required subjects to judge whether a rotated letter was forward facing or mirror reversed. Set sizes varied between two and five items. The sets were presented in a randomized order, with the constraint that a given set could not repeat until all other sets had been presented. Each set was used three times. The same scoring procedure as in the Reading Span was used.

**Stroop.** Participants were presented with a color word (red, green, or blue) presented in one of three different font colors (red, green, or blue). All words were presented in Courier New with an 18-point font. The participants’ task was to indicate the font color via key press (red = 1, green = 2, blue = 3). Participants were told to press the corresponding key as quickly and accurately as possible. Participants received 75 trials in total. Of these trials, 67% were congruent such that the word and font color matched (i.e., red printed in red), and the other 33% were incongruent (i.e., red printed in green). Congruent and incongruent trials were mixed throughout the task. The dependent measure was average incongruent trial reaction time for correct trials^2^.

**Antisaccade.** In this task (Kane et al. 2001), participants were instructed to stare at a fixation point which was onscreen for a variable amount of time (200–2200 ms). A flashing white “=” was then flashed either to the left or to the right of fixation (11.33° of visual angle) for 100 ms. This was followed by a 50 ms blank screen and a second appearance of the cue for 100 ms, making it appear as though the cue (=) flashed onscreen. Following another 50 ms blank screen, the target stimulus (a B, P, or R) appeared onscreen for 100 ms followed by masking stimuli (an H for 50 ms and an 8, which remained onscreen until a response was given). All stimuli were presented in Courier New with a 12-point font. The participants’ task was to identify the target letter by pressing a key for B, P, or R (keys left arrow, down arrow, or right arrow on the keyboard) as quickly and accurately as possible. Participants received, in order, 9 practice trials to learn the response mapping, 9 trials of the prosaccade practice, 9 trials of the Antisaccade practice, and 36 experiment trials of the Antisaccade condition. The dependent measure is the proportion of correctly identified targets.

**Psychomotor Vigilance.** In this task, participants monitor a computerized stopwatch that begins counting up in milliseconds (ms) at random intervals. The participant’s goal is to stop the counter once it begins counting by pressing a key on the keyboard. Therefore, one can measure the amount of time it takes from the onset of the counter until the time that participants stop the counter as the dependent measure. The Psychomotor Vigilance task is a simple RT task (Loh et al. 2004). Previous research has shown that it is extremely difficult to improve task performance in simple RT tasks due to their relatively basic demands on sensorimotor processes. Participants complete the Psychomotor Vigilance task for 10 min. The dependent measure is the mean of a participant’s 20% slowest trials.

**Compound Remote Associate Test.** The 30 compound remote associate (CRA) items were selected from the Bowden and Jung-Beeman (2003) normed item list. A typical CRA problem requires an individual to search through memory for a target word (“ice”) that is semantically related to three cues (“cream, skate, water”) and forms a compound word or phrase with each cue. Problems were chosen on the basis that they did not have shared cues with other items or a solution that was also a cue for another problem. Participants were given 30 s to solve each problem. For the first 5 s, the participant was unable to submit an answer. After the first 5 s, the participant was asked the likelihood they would solve the problem. After attempting all 30 problems, the participant completed a short post-experimental questionnaire, which included questions about strategies used. A participant’s score is the proportion of items correctly solved.

**TriBond.** TriBond™ is a board game developed by Mattel, Inc. (El Segundo, CA; USA) and functions similarly to the CRA. In the game, individuals are given three seemingly unrelated cues (e.g., glass, paper, aluminum) and tasked with finding the category, name, event or specific association between them (Solution: “recyclables”). Four independent raters evaluated a list of potential problems from 0 (easy) to 9 (difficult). Using averaged difficulty ratings, we selected 30 items of moderate difficulty (between 1.5 and 8.5). The flow of each problem solving trial was identical to the compound remote associates task. After attempting all 30 problems the participant completed a short post-experimental questionnaire, which included questions about strategies used. A participant’s score is the proportion of items correctly solved.

**Location Bond (LocBond).** LocBond operates similarly to CRA and Tribond. A LocBond problem consists of three clues (e.g., tower, city, French) and requires finding the target location the clues identify (Solution: Paris). We generated 30 problems where the target is a location on or in the immediate vicinity of the Arizona State University campus. The flow of each problem solving trial was identical to the compound remote associates and TriBond tasks. After attempting all 30 problems the participant completed a short post-experimental questionnaire, which included questions about strategies used. A participant’s score is the proportion of items correctly solved.

**CRA, TriBond, and LocBond Strategy.** After every CRA, TriBond, and LocBond problem the participant identified the strategy process that happened prior to submitting a solution (see Chein and Weisberg (2014), Appendix A or our Open Science Framework page for exact materials).

**Picture Source.** During the encoding phase, participants were presented with a picture (30 total pictures) in one of four different quadrants on screen for 1 s. Participants were explicitly instructed to pay attention to both the picture (item) and the quadrant it was located in (source). At test, participants were presented with 30 old and 30 new pictures in the center of the screen. Participants were required to indicate whether the picture was new or old and, if old, in what quadrant it had been presented, via keypress. Participants had 5 sec to press the appropriate key to enter their responses. A participant’s score was the proportion of correct responses.

**Cued Recall.** Participants were given three lists of 10 word pairs each. All words were common nouns, and the word pairs were presented vertically for 2 s each. Participants were told that the cue would always be the word on top and that the target would be on bottom. After the presentation of the last word, participants saw the cue word and ??? in place of the target word. Participants were instructed to type in the target word from the current list that matched the cue. Cues were randomly mixed so that the corresponding target words were not recalled in the same order as that in which they had been presented. Participants had 5 s to type in the corresponding word. A participant’s score was the proportion of items recalled correctly.

**Delayed Free Recall.** Items were presented alone for 1 s each. After a 10-item list presentation, participants engaged in a 16 s distractor task before recall: participants saw 8 three-digit numbers appear for 2 s each, and were required to type the digits in descending order (e.g., Wixted and Rohrer 1994; Unsworth 2007). At recall, participants saw three question marks appear in the middle of the screen. Participants had 45 s to recall as many of the words as possible in any order they wished from the current trial. Participants typed their responses and pressed Enter after each response clearing the screen. Prior to the practice and real trials, participants received a brief typing exercise (typing the words one-ten) to assess their typing efficiency. Participants completed 2 practice lists and 6 experiment lists. A participant’s score is the proportion of items correctly recalled.

**Category Fluency.** Participants were instructed that they should retrieve as many exemplars from the category of animals, S-words, and things of importance as possible. Each category was completed individually, and the participant was given 3 min per category (9 min total). The participants were informed that they could retrieve the exemplars in any order that they wished; they were required to type in each response, and then press Enter to record the response. We instructed the participants that they needed to keep trying to retrieve exemplars for that category throughout the entire 3 min retrieval period.

**General Knowledge.** In this task, participants complete three separate short general knowledge tests. In the first test, participants were given 10 vocabulary words and were required to select the synonym (out of five possible choices) that best matched the target vocabulary word (Hambrick et al. 1999). Participants were given unlimited time to complete the 10 items. In the second test, participants were given 10 vocabulary words and were required to select the antonym (out of five possible choices) that best matched the target vocabulary word (Hambrick et al. 1999). Participants were given unlimited time to complete the 10 items. In the third test, participants were required to answer 10 general knowledge items (e.g., What is the largest planet in our solar system? Answer: Jupiter). Participants were given unlimited time to complete the 10 items. All participants completed the synonym test first, then the antonym test, and lastly the general knowledge test. A participant’s score was the total number of items solved correctly for each test.

**Raven’s Advanced Progressive Matrices.** This test is a measure of abstract, inductive reasoning (Raven et al. 1998). Thirty-six items are presented in ascending order of difficulty. Each item consists of a display of 3 × 3 matrices of geometric patterns, arranged according to an unknown set of rules, with the bottom right pattern missing. The task is to select, among eight alternatives, the one that correctly completes the overall series of patterns. After completing two practice problems, participants had 10 min to complete the 18 odd-numbered items from the test. A participant’s score was the proportion of correct solutions. Higher scores represented better performance.

**Number Series.** In this task, subjects saw a series of numbers, arranged according to an unstated rule, and were required to induce what the next number in the series should be (Thurstone 1962). Participants selected their answer from five possible numbers that were presented. After working on five practice items, subjects had 4.5 min to complete 15 test items. A participant’s score was the proportion of items solved correctly. Higher scores represented better performance.

**Letter Sets.** In this task, participants saw five sets of four letters and were required to induce a rule that described the composition and ordering of four of the five sets (Ekstrom et al. 1976). Participants were then required to indicate the set that violated the rule. After working on two example problems, participants had 5 min to complete 20 test items. A participant’s score was the proportion of items solved correctly. Higher scores represented better performance.

### 3.1. Procedure

After consenting to participate in the experiment, participants completed the tasks in the following order. During Day 1, they completed Demographics, Reading Span, Rotation Span, Operation Span, Symmetry Span, Stroop, Antisaccade, and Psychomotor Vigilance. During Day 2, they completed Compound Remote Associates, TriBond, LocBond, Picture Source, Cued Recall, Category Fluency, General Knowledge, Raven’s Advanced Progressive Matrices, Number Series, and Letter Sets^3^.

### 3.2. Open Science and Data Screening

All experimental procedures (E-Prime), experimenter/participant notes, data files, and analysis scripts (SPSS and R) will be made available through Open Science Framework (https://osf.io/vg8mu/).

## 4. Results

Descriptive statistics for all measures can be found in Table 1^4^. As can be seen in the table, average performance mapped onto previously reported research and estimates of skew and kurtosis were at reasonable levels. Table 2 reports correlations among all dependent measures. As can be seen in Table 2, measures within a construct (i.e., WMC, attention, episodic memory, semantic memory, crystallized intelligence, fluid intelligence, and multiply-constrained problem solving) were correlated with each other.

**Figure 1:**
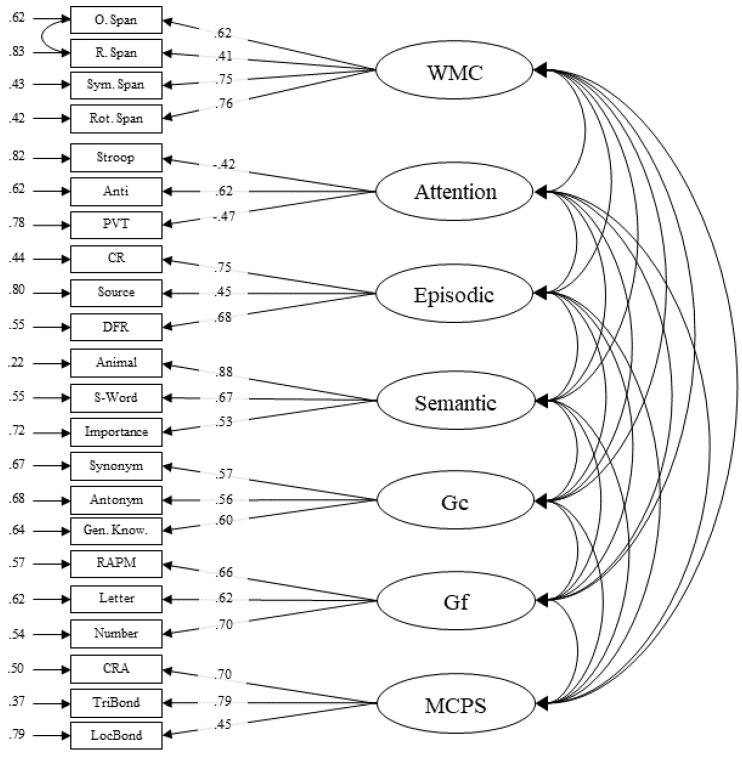
Confirmatory factor model (2) that was retained after fit comparisons. Single-headed arrows on the left-side of boxes (manifests) represent error variance. Single-headed arrows from circles (latent factors) to boxes (manifests) represent the standardized factor loadings. PVT: Psychomotor Vigilance; CR: Cued Recall; DFR: Delayed Free Recall; RAPM: Raven’s Progressive Matrices; WMC: working memory capacity; Gc: crystallized intelligence; Gf: fluid intelligence. The “A” in CRA A, TriBond A, LocBond A, and MCPS A stands for analytical, while the “I” in CRA I, TriBond I, LocBond I, and MCPS I stands for insight.

Model 1 specifies separate factors for each of the seven cognitive abilities measures in the present study (WMC, attention, episodic memory, semantic memory, crystallized intelligence, fluid intelligence, and multiply-constrained problem solving; see Figure 1). Overall, model fit was acceptable—*χ*^2^ (187) = 384.333, *p* < .001, CFI = .917, RMSEA = .048 [.041–.055]. We found significant correlations between all latent factors, but importantly a perfect (~1) correlation between the crystallized intelligence and multiply-constrained problem solving factors indicates isomorphism between these factors (see Table 3). In fact, allowing the multiply-constrained problem-solving and crystallized intelligence measures to load onto separate factors did not provide a better fit than allowing all six measures to load onto a single factor (*χ*^2^ (193) = 396.056, *p* < .001, CFI = .914, RMSEA = .048 [.041–.055], Δ*χ*^2^ (6) = 11.723, *p =* .068). Thus, in general, multiply-constrained problem solving relies heavily on crystallized intelligence. Because of that finding, we wanted to assess whether there was any *unique* variance shared among the multiply-constrained problem-solving measures, after accounting for the variance shared among all the multiply-constrained problem-solving and crystallized intelligence measures, and whether that unique variance correlated with the other cognitive factors.

**Figure 2:**
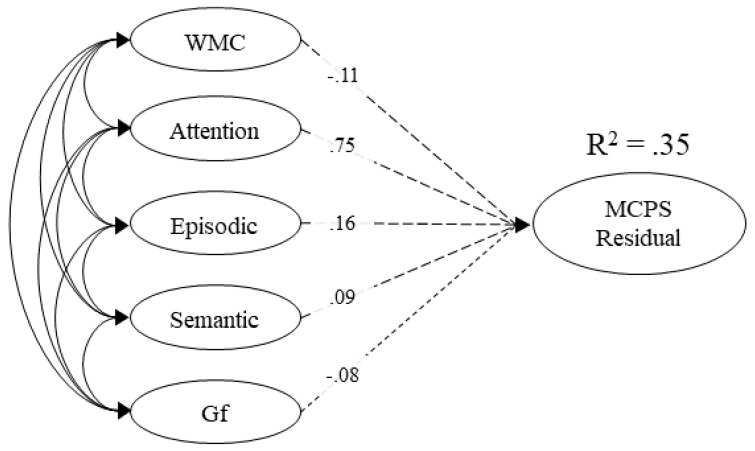
The full structural model of working memory capacity (WMC), attention control (attention), episodic memory (episodic), semantic memory (semantic), and fluid intelligence (Gf) loading on multiply-constrained problem solving residual, which is the remaining variance after accounting for shared variance between crystallized intelligence (Gc) and MCPS. Dashed lines represent nonsignificant paths. Values on paths represent standardized regression coefficients.

To do so, we specified a bi-factor model in which the three multiply-constrained problem-solving measures (CRA, TriBond, and LocBond) and the three crystallized intelligence measures (synonym, antonym, and general knowledge) were loaded onto one factor, and the three multiply-constrained problem-solving tasks were loaded onto a residual factor (MCPSr). The correlation between these factors was set to zero. Thus, the MCPSr factor represents any shared variance among the multiply-constrained problem-solving measures after accounting for the large pool of variance shared across the multiply-constrained problem-solving and crystallized factors. This factor correlated with the working memory, attention control, episodic memory, semantic memory, and fluid intelligence factors (Table 4). Thus, there is a large amount of variance in multiply-constrained problem solving that is attributable to individual differences in general and verbal knowledge. However, while we are able to account for 35% of the variance, none of the cognitive abilities account for unique variance that is not accounted for by other cognitive abilities (Figure 2).

These models represent overall performance, but a major goal of the present study was to determine whether there were task-general individual differences in the usage of analytical vs. insight solution strategies in multiply-constrained problem-solving tasks. Thus, our next set of models conditionalized accurate responses on strategy reported—that is, the number of times someone reported either an analytical solution vs. an insight solution when they correctly solved the problem. We specified factors for analytical and insight multiply-constrained problem solving separately to determine whether we could form such factors from the data, and to determine whether these factors would correlate with cognitive abilities in meaningful ways.

### 4.1. Do Answers Derived from Analytical Processes Differ from Those Found through Insight Processes at the Within-Subject Level?

Within the multiply-constrained problem solving research, there is some ambiguity about whether analytical or insight answers produce correct answers more often (see Chuderski and Jastrzebski 2018; Salvi et al. 2016 for differing results). In order to test for this difference within CRA problems, we submitted the conditionalized proportion correct (i.e., when a participant submitted a response and reported using an analytical strategy) for analytical (*M* = .376, *SD* = .282) strategies and conditionalized proportion correct for insight (*M* = .634, *SD* = .299) strategies to a paired samples *t*-test. Results indicated that when participants reported using an insight strategy, they were more often correct than when they reported using an analytics approach, *t* (434) = −14.628, *p* < .001, *d* = .701. The same paired samples *t*-test compared analytical and insight strategies for both TriBond, *t* (427) = −13.624, *p* < .001, *d* = .658, and LocBond, *t* (426) = −17.976, *p* < .001, *d* = .869. For TriBond, insight solutions (*M* = .381, *SD* = .261) were more often correct than analytical solutions (*M* = .189, *SD* = .215). Further, for LocBond, insight solutions (*M* = .634, *SD* = .246) were also found to be more often correct than analytical solutions (*M* = .338, *SD* = .259). Thus, for all three multiply-constrained problem-solving measures, insight responses were more often correct than analytical responses. Additionally, performance on the multiply-constrained problem-solving measures correlated with one another (see Table 2). This provides some indication that the tasks are likely measuring the same construct.

### 4.2. Do Different Multiply-Constrained Problem-Solving Abilities Represent Individual Task Performance or a Domain-General Ability?

**Figure 3:**
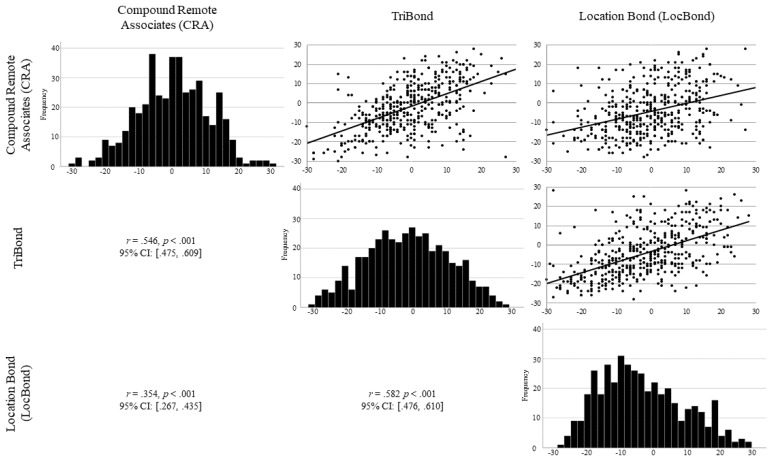
Scattermatrix with frequency distributions on the diagonal. Plotted are the difference scores for strategy usage (analytical-insight) counts (i.e., number of times a participant reported using each strategy) for the three multiply-constrained problem solving tasks (compound remote associates: CRA, TriBond, and Location Bond: LocBond).

The results of the strategy analyses indicated that when an insightful strategy is used, the response was more often correct, but the possibility existed that participants varied in the number of analytical and insight solutions reported (see Table 5 for descriptive statistics). For each participant, a difference score was computed subtracting the number of items that an insight strategy used from the number of items an analytical strategy was used without regard to accuracy. Therefore, a negative difference score indicated that a participant reported an insight strategy more often than analytical. Conversely, a positive difference score indicated that a participant reported an analytical strategy more often than insight. First, the difference scores for the three multiply-constrained problem-solving tasks correlated with one another (see Figure 3). Specifically, the strategy differences scores for CRA and TriBond were correlated, *r* (421) = .546, *p* < .001, CRA and LocBond were correlated, *r* (420) = .354, *p* < .001, and TriBond and LocBond were correlated, *r* (424) = .547, *p* < .001. The moderate-to-large positive correlations indicated that participants used strategies consistently between the three tasks. Lastly, a structural equation model was generated to determine whether a strategy usage latent factor predicted a problem solving latent factor, which consisted of the proportion of items correctly answered for the three multiply-constrained problem-solving tasks. Model fit was acceptable, *χ*^2^ (8) = 37.310, *p* < .001, CFI = .950, RMSEA = .091 [.063–.122], and strategy usage, defined as a difference score of solutions derived through analytical processes versus insight processes, was found to significantly predict problem solving performance, β = −.270, *p* < .001, accounting for 7.3% of the variance. This indicated that participants who reported using insight more often solved more multiply-constrained problems^5^.

### 4.3. Are Analytical and Insight Processes in Multiply-Constrained Problem Solving Domain General or Task Specific?

**Figure 4:**
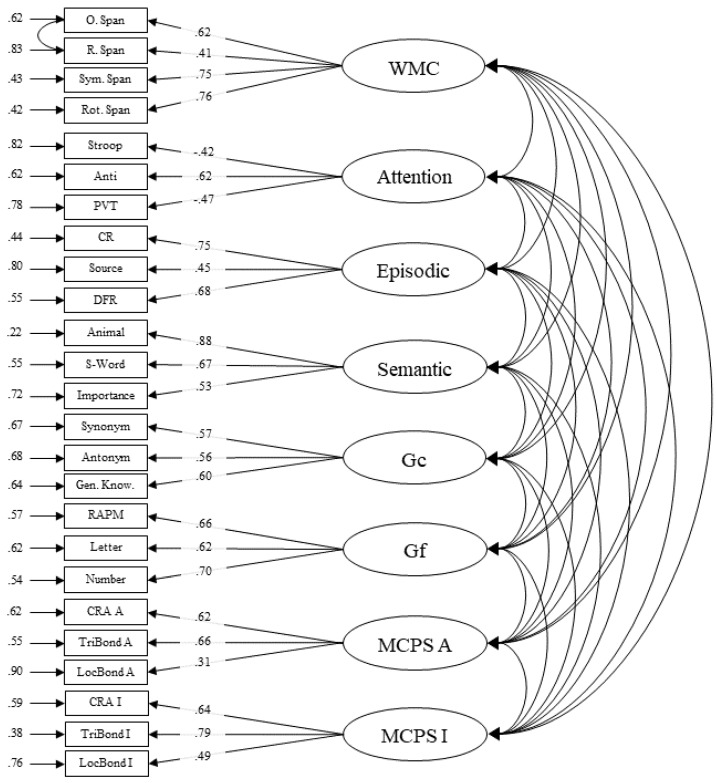
Confirmatory factor model (3) that was retained after fit comparisons. Single-headed arrows on the left-side of boxes (manifests) represent error variance. Single-headed arrows from circles (latent factors) to boxes (manifests) represent the standardized factor loadings. PVT: Psychomotor Vigilance; CR: Cued Recall; DFR: Delayed Free Recall; RAPM: Raven’s Progressive Matrices; WMC: Working Memory Capacity; Gc: crystallized intelligence; Gf: Fluid Intelligence. The “A” in CRA A, TriBond A, LocBond A, and MCPS A stands for analytical, while the “I” in CRA I, TriBond I, LocBond I, and MCPS I stands for insight.

We specified and compared two confirmatory factor analyses. Model 2 had a single multiply-constrained problem solving accuracy latent factor and Model 3 had separate latent factors for two possible solution strategies (i.e., accurate responses followed by analytical versus insight strategy response). Model 2, *χ*^2^ (253) = 530.719, *p* < .001, CFI = .879, RMSEA = .049 [.043–.055], and Model 3, *χ*^2^ (246) = 436.983, *p* < .001, CFI = .917, RMSEA = .041 [.035–.047], both had acceptable fits. The chi-square test indicated that the models were significantly different, Δ*χ*^2^ (7) = 93.736, *p* < .001, and thus the more parameterized model (Model 3) was chosen (see Figure 4). For Model 3, all latent factors were found to be significantly correlated with one another (see Table 6). Importantly, Model 3 shows that the three multiply-constrained tasks share enough common variance to form latent factors that reflect domain-general problem-solving ability. Another notable feature in Model 3 is that these individual differences in successful strategy use is also domain general in nature and these two abilities correlated.

The general trend that emerged among the latent correlations was that crystallized intelligence had the strongest correlations with multiply-constrained problem solving. Given the strength of the correlations an additional model (Model 4) was evaluated with the crystallized intelligence manifests loading onto the multiply-constrained problem solving factors and the overall model fit was acceptable, *χ*^2^ (250) = 463.260, *p* < .001, CFI = .907, RMSEA = .043 [.037–.049]. However, this model fit the data significantly worse than Model 2, Δ*χ*^2^ (4) = 26.277, *p* < .001, and Model 3 was retained.

### 4.4. Which, If Any, of the Cognitive Abilities Uniquely Predict Multiply-Constrained Problem-Solving Ability?

**Figure 5:**
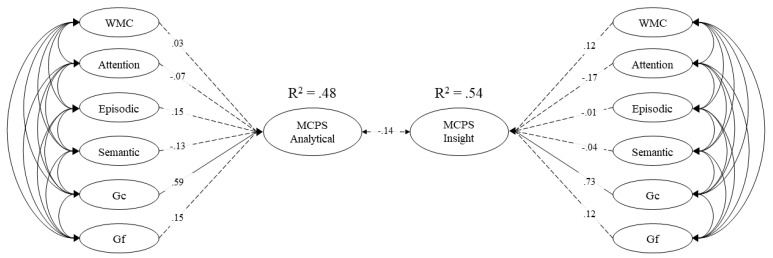
The full structural model of working memory capacity (WMC), attention control (attention), episodic memory (episodic), semantic memory (semantic), crystallized intelligence (Gc), and fluid intelligence (Gf) loading on both multiply-constrained problem solving strategies (analytical and insight). Dashed lines represent nonsignificant paths. Values on paths represent standardized regression coefficients.

Model 3 was then used to conduct a structural equation analysis to determine the predictive nature of the cognitive abilities on multiply-constrained problem solving accuracy (see Figure 5). Although the cognitive abilities (working memory, attention control, episodic memory, semantic memory, crystallized and fluid intelligence) all correlated with both multiply-constrained problem solving factors, only crystallized intelligence accounted for unique variance. Overall, the model accounted for 48% of the variance in analytical multiply-constrained problem solving and 54% of the variance in insightful multiply-constrained problem solving.

**Figure 6:**
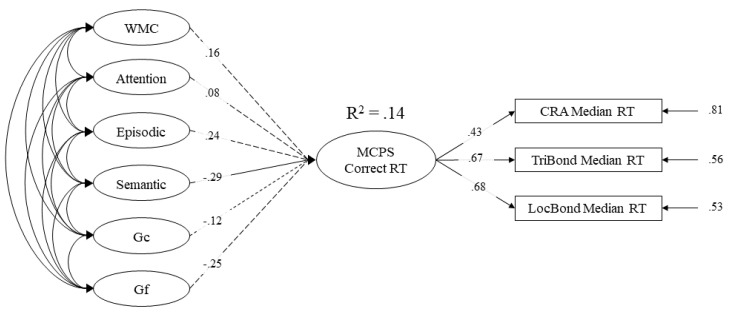
The full structural model of working memory capacity (WMC), attention control (attention), episodic memory (episodic), semantic memory (semantic), crystallized intelligence (Gc), and fluid intelligence (Gf) loading onto median response time for multiply-constrained problems correctly solved. Dashed lines represent nonsignificant paths. Values on paths represent standardized regression coefficients.

Lastly, we examined which, if any, cognitive abilities predicted the speed at which correct responses were retrieved from memory (Model 4; Figure 6). Model 4, *χ*^2^ (187) = 377.464, *p* < .001, CFI = .901, RMSEA = .047 [.040–.054] had an acceptable fit^6^. Table 7 shows latent correlations between cognitive abilities and response times for problems correctly solved (i.e., conditionalized response time). Unlike Model 2, only semantic memory and crystallized intelligence latent factors were significantly correlated with conditionalized response times. Additionally, only semantic memory was found to be a unique predictor of conditionalized response times and accounted for 14% of the variance (Figure 6).

## 5. General Discussion

The present study sought to provide the most complete picture of the underlying cognitive processes related to multiply-constrained problem solving. Before we examined each of the hypotheses related to strategy, we found that crystallized intelligence and multiply-constrained problem solving were found to be isomorphic. Our follow-up analysis examined residual variance in multiply-constrained problem solving, independent of variance accounted for by crystallized intelligence, which likely represent processes related to the problem solving process removed from having the necessary information in memory. The remaining cognitive ability latent factors were correlated with the MCPS Residual latent factor, but none were found to account for unique variance.

Our first research question asked, do answers derived from analytical processes differ from those found through insight processes at the experimental level? When a participant arrived at a solution from insight strategies, that answer was more often correct than when it was derived from analytical processes. Second, we asked, do multiply-constrained problem-solving abilities represent individual task performance or domain-general ability? Results indicated that there exists a domain general multiply-constrained problem-solving ability. Next, we asked, are analytical and insight processes in multiply-constrained problem solving domain general or task specific? The best-fitting model contained latent factors for each of the cognitive abilities measured (working memory, attention control, episodic memory, semantic memory, crystallized and fluid intelligence) and two multiply-constrained problem solving latent factors (analytical and insight). The structural equation analysis accounted for 48% of the variance in analytical multiply-constrained problem solving and 54% of the variance in insightful multiply-constrained problem solving. Lastly, which, if any, of the cognitive abilities uniquely predicts multiply-constrained problem solving? The structural model indicated that each of the underlying cognitive abilities was correlated with the problem solving latent factors, only crystallized intelligence was found to have unique predictive value. Relatedly, when cognitive abilities were used to predict multiply-constrained problem solving correct response times only, semantic memory contributed unique predictive value. This pair of findings indicate that different, but related, cognitive abilities account are necessary for multiply-constrained problem solving.

Across all three tasks, our data demonstrate that answers retrieved through insight processes are more often correct than analytical strategies. Of primary importance is that the sets of strategy solutions between multiply-constrained problem-solving tasks load onto unique factors, which indicates that strategy processes are domain general rather than task specific. While there is a debate to be had whether this is evidence for the special-processes view of insight (see Weisberg (2015) for a thorough review), it is our opinion that differences in strategy reporting are an issue of phenomenological sensation and perception. Specifically, any retrieved answer is always going to contain an “Aha”-like sensation and the strength of that sensation may be related to the associative strength between cues and retrieved targets of the solver. Additionally, participants are instructed to solve as many problems as possible and if a solver wants to achieve best performance, any retrieved answer, regardless of how the solver becomes consciously aware of the answer, should be compared against each cue to ensure its accuracy. Therefore, all retrieved answers should employ both analytical and insight strategies.

One of the most consistent findings in the compound remote associates literature is its relation to working memory (Wiley and Jarosz 2012b). Our data replicate the previous relation between working memory and CRA and establishes a similar positive relation to the other multiply-constrained problem tasks (TriBond and LocBond). However, unlike previous literature, we find that working memory is not a unique predictor of multiply-constrained problem solving (Chuderski and Jastrzebski 2018; Kane et al. 2004). This may be due to our creation of a multiply-constrained problem solving factor rather than grouping it with other more common divergent (e.g., alternative uses) or convergent (e.g., “dot” problem) tasks. Moreover, we replicate the known positive relation between CRA and Antisaccade (*r* = .23 analytical and *r* = .24 insight), and no relation with the Stroop task (Chein and Weisberg 2014; Chuderski and Jastrzebski 2018). If the Antisaccade is a measure of inhibition, albeit the inhibition of a physical movement, it is not surprising to find it related to multiply-constrained problem solving. Gupta et al. (2012) demonstrated that an individual will perform better on CRA problems when they can avoid prepotent or high-frequency candidate answers. It could be that individuals who are better at multiply-constrained problem solving are better at delaying the submission of spontaneously retrieved answers and waiting to confirm it is the correct answer.

Our data indicate a small, but largely consistent, correlation between tasks designed to measure episodic memory and multiply-constrained problem solving, which to our knowledge has not been previously found in this literature. For both the source memory and cued recall tasks, the participant is shown a cue from which they must retrieve information stored in memory. Therefore, it logically follows that they should be related to multiply-constrained problem solving, which are tasks where the participant is shown cues and asked to retrieve information stored in memory. More specifically, the associative binding or processes engaged during encoding and retrieval of episodic memories (see Cox and Criss 2017; Kahana et al. 2008 for a review) may be similarly engaged during multiply-constrained problem solving. For example, while attempting a LocBond problem, the solver may engage in a mental walk through the location they believe the target to be located (participants did report engaging in mental walks on opened ended questions at the end of the task). Previous work by Davelaar (2015) and Smith et al. (2013) demonstrated that semantic search is related to multiply-constrained problem solving. Additionally, Lee and Therriault (2013) found performance on fluency tasks was predictive of compound remote associate problem-solving ability. Our data largely replicate the previous literature. However, despite strong correlations between the three fluency tasks, the three fluency tasks do not consistently correlate with multiply-constrained problem solving.

To date, several researchers have identified that measures of fluid intelligence are related to the compound remote associates (Chermhini et al. 2012; Chuderski 2014; Chuderski and Jastrzebski 2018; Kane et al. 2004; Lee and Therriault 2013). We replicate the previous literature and extend the finding to the novel TriBond and LocBond tasks. However, unlike the recent findings of Chuderski and Jastrzebski (2018), fluid intelligence did not account for unique variance, but this may be partially due to differences in tasks used to measure reasoning ability (they used Raven’s, Figural Analogies, Number Series, and Logic Problems) and our inclusion of other cognitive measures which may have shared variance with fluid intelligence. Additionally, the difference in latent correlations between problems solved and Gf with analytical strategies and insight strategies is present in both experiments.

To date, there has been discussion about the nature of crystallized and fluid intelligence in creativity, and compound remote associates by proxy (Benedek and Fink 2019; Cortes et al. 2019; Marko et al. 2018; Kim 2005; Silvia 2015). Replicating Lee and Therriault (2013) we find a positive relation between measures of intelligence and problem-solving ability. Additionally, our data indicate that crystallized intelligence was the only latent factor to offer unique predictive value. Moreover, model comparisons indicate that multiply-constrained problems are different from measures of verbal and general knowledge. Individuals who perform better on measures of crystallized intelligence may have a flatter (greater ability to access both frequent and infrequent associations) and more interconnected semantic network, in addition to stronger associations between what are traditionally weakly associated cues and targets. Network analyses (see Kenett and Faust 2019) between individuals of low and high crystallized intelligence may provide further elucidation on the relation between verbal knowledge and multiply-constrained problem solving. Alternatively, using tasks such as TriBond and LocBond, which require more specific areas of knowledge, may have increased the relation between crystallized intelligence and multiply-constrained problem solving.

One possible limitation is that both TriBond and LocBond are new experimental tasks that have not been as rigorously validated as the CRA. Specifically, the range and control over the difficulty of the problems may not be as strong as it is for the CRA. Additionally, the LocBond problems for this experiment were specifically designed for the population the participants were recruited from and may perform differently with other participant samples. However, LocBond items that are less population specific can be easily generated. For example, domain general LocBond items could ask about popular tourist destinations (e.g., the Eiffel tower, Disneyland, Mt. Rushmore). However, the fact that the three tasks loaded well onto factors (analytical and insight) gives us confidence that these tasks tapped into a common cognitive ability.

Additionally, the use of a self-report measure for strategy usage could represent a proxy of item difficulty. Specifically, when an item is more difficult, and the solution is not being retrieved with ease, the participant may be more likely to report using an analytical approach. Specifically, one way to conceptualize these responses is as a type of metacognitive assessment of the emergence of the solution into mind. That assessment, like all metacognitive assessments, is governed by multiple inputs including features of the problem (i.e., difficulty), features of the problem solver (i.e., cognitive abilities), and features of the problem solving context (i.e., solving a single problem or a large set of problems). Therefore, these judgements are almost certainly not process (strategy) pure. They likely reflect some combination of a retrospective evaluation of strategy usage plus other inputs which may or may not actually be related to the problem solving approach (Ball et al. 2014). Future research could utilize alternative measures (e.g., EEG or MRI) to obtain indirect measures of strategy usage.

In the future, an evaluation of whether these multiply-constrained problem-solving tasks share any cognitive underpinnings with other measures of creativity should be conducted as since its conception the (compound) remote associates task has been linked to both creativity and insight. Previous work has shown for the CRA how and when insight responses are submitted is not always the same (Cranford and Moss 2012). Others have noted that the CRA does not load onto factors with other insight tasks (Chuderski and Jastrzebski 2018; Lee and Therriault 2013; Lee et al. 2014). Our findings seem to indicate why this was the case. As noted, individuals do not always reach impasse when solving a CRA problem (Cranford and Moss 2012) and can intuitively arrive at the solution within seconds of seeing the cues (Bolte and Goschke 2005; Topolinksi and Strack 2009). Additionally, given our model comparisons, while the multiply-constrained problem-solving tasks are strongly related to crystallized intelligence measures there is not complete overlap. Not present in our experiment are common creativity measures (e.g., Alternative Uses). As both sets of tasks utilize both convergent and divergent processes and share strong relations to intelligence, it is difficult to predict whether multiply-constrained problem solving and creativity will remain as unique factors or whether a combined creativity and multiply-constrained problem solving latent factor will emerge due to their shared underpinnings and processes. Therefore, an experiment that contains multiply-constrained problem solving, intelligence, and commonly used measures of creativity and insight should be conducted to fully assess the variance-covariance structure of these constructs.

As with any experimental or laboratory task, the question must be asked, how do the current results map onto behavior in the *real world*? The answer is *Jeopardy!* In 2014, their “Battle of the Decades” aired and, late into the tournament, the category “Common Bonds” appeared. The first clue in the category read “cupid, dancer, prancer” and within moments, the correct response “reindeer” was produced, and thus science and life intersected. The contestant who responded correctly could not have done so had they not known any of those names or that they shared a connection with reindeer. More specifically, despite the demands on effective goal maintenance, attention control, memory search efficiency, and problem solving skill, one cannot retrieve an answer from memory if the answer does not already reside there.

## Figures and Tables

**Table 1:** Descriptive statistics for each dependent measure. For the compound remote associates (CRA), TriBond, and Location Bond (LocBond) data shown are for unconditionalized (overall) and conditionalized (analytical and insight) proportion correct. Response times (RT) are for correct responses only.

Task	N	Min.	Max.	Mean	Std. Dev.	Skew.	Kurt.	α
Reading Span	459	17	75	54.76	11.18	−0.65	0.09	0.78
Operation Span	456	0	75	56.11	15.84	−1.42	1.55	0.89
Symmetry Span	458	2	42	26.88	8.87	−0.56	−0.21	0.82
Rotation Span	454	3	72	35.28	13.47	−0.06	−0.49	0.87
Stroop (Incongruent)	459	464.30	1989.82	895.51	242.38	1.29	2.16	0.89
Antisaccade	458	.16	.98	0.66	0.17	−0.53	−0.37	0.84
Psychomotor Vigilance	457	277.06	477.79	369.90	37.01	0.18	−0.61	0.99
CRA Overall Accuracy	435	.00	.87	0.35	0.16	−0.02	−0.15	0.73
CRA RT (median)	426	1065	21795	4807.40	2579.06	1.56	4.98	−
Strategy—Analytical	435	.00	1.00	0.38	0.28	0.47	−0.73	−
Strategy—Insight	435	.00	1.00	0.63	0.30	−0.69	−0.47	−
TriBond Overall Accuracy	428	.00	.77	0.21	0.14	0.78	0.33	0.79
TriBond RT (median)	409	1039	19112	5626.20	2938.99	1.52	3.20	−
Strategy—Analytical	428	.00	1.00	0.19	0.21	1.41	2.05	−
Strategy—Insight	428	.00	1.00	0.38	0.26	0.37	−0.63	−
LocBond Overall Accuracy	427	.00	.73	0.40	0.14	−0.13	−0.40	0.74
LocBond RT (median)	426	1345	13440	5061.40	2045.68	1.22	1.94	−
Strategy—Analytical	427	.00	1.00	0.34	0.26	0.37	−0.57	−
Strategy—Insight	427	.00	1.00	0.63	0.24	−0.75	0.34	−
Picture Source	434	.00	1.00	0.73	0.19	−1.05	1.22	0.85
Cued Recall	415	.00	.97	0.35	0.22	0.65	−0.24	0.87
Delay Free Recall	414	.00	.92	0.43	0.19	−0.04	0.19	0.88
Fluency—Animal	432	.00	72.00	35.30	10.55	0.00	0.56	−
Fluency—S-Word	432	11.00	74.00	40.09	10.57	0.27	−0.06	−
Fluency—Importance	432	4.00	61.00	27.45	10.90	0.60	0.03	
Synonym	433	.00	.90	0.31	0.18	0.69	0.18	0.45
Antonym	433	.00	.90	0.35	0.18	0.49	−0.09	0.34
General Knowledge	433	.00	1.00	0.49	0.22	0.07	−0.58	0.58
Raven’s Prog. Matrices	267	.06	.94	0.47	0.19	−0.08	−0.44	0.75
Number Series	209	.07	1.00	0.61	0.18	−0.16	−0.48	0.71
Letter Sets	216	.10	.90	0.51	0.17	−0.13	−0.49	0.70

**Table 2:** Correlations between dependent measures.

		**1.**	**2.**	**3.**	**4.**	**5.**	**6.**	**7.**	**8.**	**9.**	**10.**	**11.**	**12.**	**13.**	**14.**	**15.**
1.	Operation Span															
2.	Reading Span	0.49														
3.	Symmetry Span	0.49	0.25													
4.	Rotation Span	0.44	0.32	0.57												
5.	Stroop (Incong.)	−0.22	−0.06	−0.23	−0.20											
6.	Antisaccade	0.19	0.19	0.24	0.28	−0.14										
7.	Psych. Vigilance	−0.16	−0.11	−0.16	−0.13	0.31	−0.31									
8.	Picture Source	0.09	0.09	0.23	0.26	−0.15	0.20	−0.10								
9.	Cued Recall	0.13	0.21	0.15	0.21	−0.02	0.13	0.02	0.34							
10.	Delay Free Recall	0.10	0.28	0.22	0.27	−0.08	0.18	−0.05	0.24	0.52						
11.	Fluency—Animal	0.13	0.20	0.19	0.14	−0.09	0.17	−0.06	0.17	0.28	0.29					
12.	Fluency—S-Word	0.11	0.23	0.18	0.17	−0.14	0.13	−0.06	0.21	0.21	0.21	0.58				
13.	Fluency—Import.	0.09	0.09	0.12	0.05	−0.04	−0.03	0.04	0.09	0.12	0.09	0.47	0.41			
14.	Synonym	0.12	0.26	0.09	0.03	−0.09	0.19	−0.06	0.10	0.25	0.20	0.27	0.20	0.09		
15.	Antonym	0.12	0.20	0.08	0.06	0.00	0.17	−0.09	0.17	0.32	0.27	0.28	0.21	0.05	0.38	
16.	General Knowledge	0.11	0.15	0.11	0.05	−0.14	0.22	−0.17	0.12	0.17	0.17	0.29	0.17	0.02	0.33	0.30
17.	Raven’s	0.20	0.18	0.21	0.19	−0.20	0.33	−0.13	0.33	0.24	0.23	0.17	0.14	−0.02	0.23	0.20
18.	Number Series	0.32	0.15	0.28	0.31	−0.19	0.31	−0.13	0.13	0.26	0.20	0.23	0.07	−0.01	0.25	0.29
19.	Letter Sets	0.21	0.19	0.18	0.21	−0.16	0.28	−0.03	0.18	0.22	0.30	0.28	0.19	0.06	0.15	0.23
20.	CRA (Overall)	0.18	0.29	0.22	0.14	−0.14	0.35	−0.15	0.22	0.32	0.31	0.36	0.30	0.05	0.38	0.41
21.	CRA (RT)	−0.13	−0.18	−0.05	−0.09	0.10	−0.10	−0.05	−0.01	−0.07	−0.08	−0.20	−0.22	−0.13	−0.10	−0.07
22.	CRA (Analytical)	0.11	0.15	0.12	0.08	−0.02	0.24	−0.02	0.17	0.23	0.23	0.16	0.15	0.04	0.19	0.18
23.	CRA (Insight)	0.13	0.24	0.15	0.13	−0.06	0.23	−0.13	0.09	0.22	0.23	0.19	0.13	−0.02	0.26	0.28
24.	TriBond (Overall)	0.14	0.26	0.11	0.12	−0.08	0.27	−0.12	0.25	0.35	0.30	0.40	0.27	0.05	0.43	0.41
25.	TriBond (RT)	−0.01	−0.08	0.05	0.06	0.01	−0.01	−0.02	0.10	−0.04	−0.01	−0.11	0.01	−0.06	−0.05	−0.05
26.	TriBond (Analytical)	0.09	0.15	0.09	0.13	−0.10	0.16	−0.02	0.21	0.22	0.18	0.17	0.17	0.05	0.30	0.25
27.	TriBond (Insightl)	0.08	0.20	0.08	0.10	−0.03	0.18	−0.08	0.14	0.29	0.20	0.30	0.19	0.07	0.29	0.31
28.	LocBond (Overall)	0.12	0.13	0.08	0.05	0.01	0.07	−0.04	0.08	0.26	0.20	0.22	0.11	0.06	0.24	0.28
29	LocBond (RT)	−0.08	−0.09	0.08	0.15	0.07	0.01	0.01	0.12	0.03	0.00	−0.18	−0.04	−0.10	−0.08	−0.07
30.	LocBond (Analytical)	0.02	0.06	0.00	−0.03	−0.01	0.09	−0.08	0.04	0.14	0.09	0.06	−0.04	−0.02	0.15	0.11
31.	LocBond (Insight)	0.14	0.13	0.10	0.15	0.05	0.07	0.00	0.08	0.23	0.14	0.16	0.09	0.04	0.17	0.19
		**16.**	**17.**	**18.**	**19.**	**20.**	**21.**	**22.**	**23.**	**24.**	**25.**	**26.**	**27.**	**28.**	**29.**	**30.**
1.	Operation Span															
2.	Reading Span															
3.	Symmetry Span															
4.	Rotation Span															
5.	Stroop (Incong.)															
6.	Antisaccade															
7.	Psych. Vigilance															
8.	Picture Source															
9.	Cued Recall															
10.	Delay Free Recall															
11.	Fluency—Animal															
12.	Fluency—S-Word															
13.	Fluency—Import.															
14.	Synonym															
15.	Antonym															
16.	General Knowledge															
17.	Raven’s	0.24														
18.	Number Series	0.25	0.40													
19.	Letter Sets	0.16	0.39	0.50												
20.	CRA (Overall)	0.47	0.37	0.39	0.39											
21.	CRA (RT)	−0.07	0.02	−0.13	−0.10	−0.12										
22.	CRA (Analytical)	0.24	0.25	0.19	0.20	0.48	0.11									
23.	CRA (Insight)	0.30	0.27	0.18	0.23	0.62	−0.03	0.20								
24.	TriBond (Overall)	0.58	0.41	0.35	0.31	0.57	−0.09	0.33	0.41							
25.	TriBond (RT)	−0.05	0.09	−0.10	−0.21	−0.06	0.31	0.07	0.04	−0.02						
26.	TriBond (Analytical)	0.29	0.29	0.16	0.14	0.32	−0.03	0.42	0.21	0.54	0.12					
27.	TriBond (Insightl)	0.38	0.29	0.19	0.21	0.38	−0.05	0.20	0.50	0.70	−0.01	0.26				
28.	LocBond (Overall)	0.36	0.28	0.22	0.18	0.27	−0.04	0.11	0.18	0.38	−0.02	0.17	0.26			
29	LocBond (RT)	−0.11	0.05	−0.11	−0.09	−0.03	0.27	0.05	0.04	−0.02	0.46	0.12	0.02	−0.08		
30.	LocBond (Analytical)	0.19	0.19	−0.02	0.06	0.15	−0.03	0.22	0.04	0.16	0.00	0.17	0.11	0.40	0.09	
31.	LocBond (Insight)	0.23	0.24	0.18	0.14	0.17	−0.03	0.02	0.30	0.27	0.06	0.06	0.40	0.55	0.03	0.10

Note: If correlation greater than .19 or less than −.19 significant at *p* < .05 using Hochberg False Discovery Rate correction.

**Table 3:** Latent factor correlations between cognitive abilities and multiply-constrained problem solving (MCPS).

		1.	2.	3.	4.	5.	6.
1.	Working Memory						
2.	Attention Control	.56					
3.	Episodic Memory	.43	.31				
4.	Semantic Memory	.28	.25	.44			
5.	Crystalized Intelligence	.21	.48	.53	.51		
6.	Fluid Intelligence	.53	.70	.59	.33	.54	
7.	MCPS	.27	.50	.61	.52	1	.72

Note: All correlations significant at *p* < .001, except working memory and crystalized intelligence, *p* < .01.

**Table 4:** Latent factor correlations between working memory capacity (WMC), attention control (Att), episodic memory (Epi), semantic memory (Sem), crystallized intelligence (Gc), fluid intelligence (Gf), and multiply-constrained problem solving residual (MCPS Residual).

	WMC	Att	Epi	Sem	Gf
MCPS Residual	.22	.44	.55	.50	.63

Note: All correlations significant at *p* < .001.

**Table 5:** Descriptive statistics for each multiply-constrained problem-solving task (compound remote associate: CRA, TriBond, and Location Bond: LocBond). The total number of times people reported each strategy (analytical and insight) are reported for each task. The difference score is the number of analytical responses minus the number of insight responses (A—I).

Task (Strategy)	N	Min.	Max.	Mean	Std. Dev.	Skew.	Kurt.
CRA (Analytical)	435	0.00	30.00	10.62	5.92	0.37	−0.26
CRA (Insight)	435	0.00	30.00	10.77	5.89	0.43	−0.07
CRA Difference (A—I)	435	−30.00	30.00	−0.15	10.62	−0.06	−0.20
TriBond (Analytical)	428	0.00	28.00	10.25	6.59	0.33	−0.59
TriBond (Insight)	428	0.00	30.00	12.11	6.73	0.26	−0.54
TriBond Difference (A—I)	428	−30.00	28.00	−1.86	12.33	0.01	−0.64
LocBond (Analytical)	427	0.00	29.00	8.98	6.66	0.59	−0.43
LocBond (Insight)	427	0.00	28.00	13.22	6.49	−0.16	−0.62
LocBond Difference (A—I)	427	−28.00	28.00	−4.25	12.37	0.43	−0.58

**Table 6:** Latent factor correlations between working memory capacity (WMC), attention control (Att), episodic memory (Epi), semantic memory (Sem), crystallized intelligence (Gc), fluid intelligence (Gf), and multiply-constrained problem solving (MCPS).

	WMC	Att	Epi	Sem	Gc	Gf	MCPS-A
MCPS—Analytical	.22	.35	.49	.29	.66	.48	
MCPS—Insight	.23	.32	.44	.36	.72	.45	.43

Note: All correlations significant at *p* < .001, except working memory and crystalized intelligence, *p* < .01.

**Table 7:** Latent factor correlations between cognitive abilities and median response times for multiply-constrained problems correctly solved (MCPS RT).

	WMC	Attention	Episodic	Semantic	Gc	Gf
MCPS RT	.07	−.06	.00	−.27 **	−.19 **	−.13

Note: ** = *p* < .01.

## Data Availability

Data is contained within the article or supplementary material The data presented in this study are available in Open Science Framework (https://osf.io/vg8mu/).

## Footnotes

1 The fluid intelligence tasks were added approximately halfway through data collected. Thus, not all participants completed the fluid intelligence tasks. Fluid intelligence remained in the models as prior to data collection, we had planned to use full information maximum likelihood (FIML) data procedures to account for missing data. Additionally, models were run with the fluid intelligence tasks removed and the general pattern of results remained the same. Specifically, model fit was acceptable and crystallized intelligence remained the only unique predictor.

2 There are two measures that can be derived from the Stroop task as an individual difference: the difference in RT between incongruent and congruent trials, and the average RT for incongruent trials. In our data, the reliability for the difference score was low (0.626), so we used the incongruent RT score, which had higher reliability and higher correlations with our other two attention control tasks.

3 The order of tasks remained constant across all participants. Because we were testing participants in two-hour sessions across two days, we wanted any task-order effects to be constant for all participants.

4 All analyses were replicated only using data from those who participated after the fluid intelligence tasks were added to the study. Tables and models can be found in supplemental materials. Of critical importance, the underlying structure of the relations between latent factors are consistent between the two sets of analyses. Therefore, we have decided to present the findings of the larger data set.

5 The curious reader may be interested in the relations between cognitive abilities (working memory capacity, attention control, episodic and semantic memory, and crystallized and fluid intelligence) and strategy reporting. We fit a model with a strategy usage latent factor and assessed whether those cognitive abilities predicted strategy usage. This exploratory analysis produced a model with overall acceptable fit, *χ*^2^ (187) = 336.703, *p* < 0.001, CFI = 0.926, RMSEA = 0.042 [0.035–0.049]. However, no cognitive ability was shown to be a unique predictor of strategy reporting. Additionally, the model accounted for only 6.3% of the variance in strategy usage, which indicates that strategy adoption may be fairly independent of cognitive abilities.

6 Upon reviewer request, we examined another likely model that included strategy usage as a latent factor predicting correct response times. This exploratory analysis produced a model with overall acceptable fit, *χ*^2^ (246) = 433.992, *p* < 0.001, CFI = 0.916, RMSEA = 0.041 [0.034–0.047]. The strategy usage latent factor did not correlate with responses times or emerge as a unique predictor.

## Appendix A

### Appendix A.1. Instruction Screen 1

We are also interested in finding out how you solved each problem. Problems can be solved in two general ways: as the result of ANALYTICAL or as the result of a sudden INSIGHT. Solving the problem by an ANALYTICAL means that when you first thought of the word, you did not know whether it was the answer, but after thinking about it analytically (for example, trying to combine the single word with each of the three problem words), you figured out that it was the answer. Solving the problem by a sudden INSIGHT means that as soon as you thought of the word, you knew that it was the answer. The solution word came with a feeling that it was correct (“It popped into my head”; “Of course!”; “I had an Aha!”).

### Appendix A.2. Instruction Screen 2

After you solve each problem, you will be asked whether you solved it insightfully or strategically. You will see a rating scale with numbers 1–4.

The rating 1 will be marked “COMPLETE Analytical.” Marking a rating of 1 means that when you thought of the word, at first you did not know whether it was the answer, but after thinking about it strategically (for example, trying to combine the single word with each of the three problem words) you figured out that it was the answer.

A rating of 2 will be marked “PARTIAL Analytical.” A rating of 2 means that you did not immediately know the word was the answer, but you did not have to think about it much either. For example, after figuring out how the solution went with the first two stimulus words, you realized that it was the solution.

The rating of 3 will be marked “PARTIAL Insight.” Choosing a rating of 3 means that you had a weaker feeling of insight (not as strong as a rating of 4): you felt that the word you thought of might have been the answer, but it was not as obvious as “Of course!” You might have had to check the solution with one of the words to make sure it was correct.

The rating of 4 will be marked “COMPLETE Insight.” A rating of 4 means that as soon as you thought of the word you knew that it was the answer; the solution word came with a feeling that it was correct (“It popped into my head”; “Of course!”; “I had an Aha!”).

It is up to you to decide what rating to give. There are no right or wrong answers.
